# Identification of Key Genes and Imbalanced SNAREs Assembly in the Comorbidity of Polycystic Ovary Syndrome and Depression

**DOI:** 10.3390/genes15040494

**Published:** 2024-04-15

**Authors:** Yi Cao, Weijing Wang, Xuxia Song, Qian Wen, Jing Xie, Dongfeng Zhang

**Affiliations:** 1 Biomedical Center, Qingdao University, No. 308 Ningxia Road, Qingdao 266021, China; qddxcaoyi@qdu.edu.cn (Y.C.); sxx688@163.com (X.S.); wenqian7777@163.com (Q.W.); 2 Department of Epidemiology and Health Statistics, Public Health College, Qingdao University, No. 308 Ningxia Road, Qingdao 266021, China; wangwj@qdu.edu.cn

**Keywords:** polycystic ovary syndrome (PCOS), depression, comorbidity, SNAREs, single nucleotide polymorphisms (SNPs)

## Abstract

Background: Women with polycystic ovary syndrome (PCOS) have increased odds of concurrent depression, indicating that the relationship between PCOS and depression is more likely to be comorbid. However, the underlying mechanism remains unclear. Here, we aimed to use bioinformatic analysis to screen for the genetic elements shared between PCOS and depression. Methods: Differentially expressed genes (DEGs) were screened out through GEO2R using the PCOS and depression datasets in NCBI. Protein–protein interaction (PPI) network analysis and enrichment analysis were performed to identify the potential hub genes. After verification using other PCOS and depression datasets, the associations between key gene polymorphism and comorbidity were further studied using data from the UK biobank (UKB) database. Results: In this study, three key genes, namely, *SNAP23*, *VTI1A*, and *PRKAR1A*, and their related SNARE interactions in the vesicular transport pathway were identified in the comorbidity of PCOS and depression. The rs112568544 at *SNAP23*, rs11077579 and rs4458066 at *PRKAR1A*, and rs10885349 at *VTI1A* might be the genetic basis of this comorbidity. Conclusions: Our study suggests that the *SNAP23*, *PRKAR1A*, and *VTI1A* genes can directly or indirectly participate in the imbalanced assembly of SNAREs in the pathogenesis of the comorbidity of PCOS and depression. These findings may provide new strategies in diagnosis and therapy for this comorbidity.

## 1. Introduction

Polycystic ovary syndrome (PCOS) is a frequently occurring endocrine disorder with a worldwide prevalence of 5–10% in women of reproductive age [1]. Characterized by hyperandrogenemia, chronic anovulation, and polycystic ovary morphology, PCOS is associated with some endocrine and energy metabolic disorders, such as obesity, dyslipidemia, insulin resistance (IR), type 2 diabetes, and cardiovascular disease [2]. Moreover, increased depression is also observed in PCOS, significantly deteriorating the quality of life of affected females [3]. It is reported that depression affects between 28% and 64% of patients with PCOS [4], and a three to eight times higher prevalence of depressive symptom has been found in women with PCOS than in control groups [5]. In addition, the literature also suggests an overlap of clinical symptoms between PCOS and depression [6]. Accordingly, as recommended in the 2018 evidence-based guidelines on PCOS management, the International PCOS Network advised that depression should be routinely screened for in all adolescents and women with PCOS at the time of diagnosis [7].

Multiple possible associations or shared links are mentioned between PCOS, PCOS related metabolic disorders, and depression [1,8,9], which indicate that rather than merely considering depression as a consequence of PCOS, the relationship between PCOS and depression is more likely to be comorbid. Studies have demonstrated that PCOS is a pro-inflammatory state, characterized by increased levels of pro-inflammatory markers [10]. Meanwhile, depression is also evidenced to be an inflammatory disorder with increased levels of inflammatory markers [11]. Thus, there is a possibility of an inflammatory link existing between PCOS and depression. Aside from the inflammatory link, several studies have suggested an association between obesity, IR, and hyperandrogenism observed in both PCOS and depression [1,8,12,13,14], indicating the possibility of an interconnection between PCOS and depression. Hence, depression might share common genetic components, such as differentially expressed genes (DEGs) with PCOS to cause comorbidity, despite gaps in the literature to date.

Additionally, scattered in both coding and regulatory regions throughout the genome, single nucleotide polymorphisms (SNPs) are the most common type of genetic variation. The SNPs remain stable genetically and are proven to be associated with the pathogenesis of diseases, making them biological markers to identify diseases [15]. However, the genetic variation underlying the comorbidity of PCOS and depression has not yet been elucidated.

Considering that the DEGs between PCOS and depression were unclear, in our study we first tried to determine the DEGs shared between PCOS and depression with bioinformatic analysis using data from the GEO database in NCBI, and then verified the key genes using genetic variation data from the UK biobank (UKB) database. This study may help to understand the molecular mechanism of the comorbidity of PCOS and depression and provide a new strategy for diagnosis and therapy.

## 2. Materials and Methods

### 2.1. Data Acquisition

The mRNA expression profile datasets GSE8157 and GSE23848 were downloaded from the GEO database in NCBI (https://www.ncbi.nlm.nih.gov/geo/ accessed on 10 September 2023) in the format of Series Matrix File(s). The dataset GSE8157 which was used to screen the DEGs on PCOS was hosted on the GPL570 platform [(HG-U133_Plus_2) Affymetrix Human Genome U133 Plus 2.0 Array]. The participants in GSE8157 dataset were from Denmark and the criteria of participant selection included the following: (1) Patients were diagnosed as PCOS with the criteria to have irregular menstrual periods with cycle length > 35 days during the last year, free testosterone level above reference interval (>0.035 nmol/L), and/or hirsutism (total Ferriman-Gallwey score > 7). (2) All patients with PCOS accepted to withdraw from oral contraceptives > 3 months before evaluation and consented to use barrier contraception combined with spermatocidal cream during the study period. Participants were excluded if they were pregnant. (3) Women with diabetes, hypertension, elevated liver enzyme levels, adrenal enzyme defects, hyperprolactinemia, and hypothyroidism were excluded from the study. The dataset GSE23848 that contained data on patients with depression and healthy controls was located on the GPL6106Sentrix Human-6 v2 Expression BeadChip platform. The participants in the GSE23848 dataset were from USA and the criteria of participant selection included the following: (1) Age between 18 and 65 years, diagnosis of BPD, currently depressed as defined by the DSM-IV-TR (Diagnostic and Statistical Manual of Mental Disorders), and not currently being treated with lithium. Study eligibility was based on clinical diagnosis rather than any predetermined severity criteria. (2) DSM-IV-TR diagnoses other than BPD-I or BPD-II and current or recent abuse of illicit substances (verified by urine toxicology screening) were excluded. Raw data were log_2_-transformed and quantile-normalized prior to analysis.

### 2.2. Screening of DEGs

The annotated probes in the datasets were analyzed using GEO2R (https://www.ncbi.nlm.nih.gov/geo/geo2r/ accessed on 10 September 2023) with the GeoQuery and Limma R packages. The normalized expression matrix from the microarray data was represented by a box line plot. DEGs were screened with the cut-off criteria as *p*-value < 0.05 and |log_2_FC| ≥ 0.263 (1.2 folder). Consistent DEGs between the GSE8157 and GSE23848 datasets were identified by R software (version 4.0.3) and visualized through a Venn diagram (http://bioinformatics.psb.ugent.be/webtools/Venn/ (accessed on 10 September 2023)).

### 2.3. Protein–Protein Interaction (PPI) Network Analysis and Identification of Hub-Genes

PPI network analysis was performed using the STRING database (https://string-db.org/ accessed on 15 September 2023) with the minimum interaction score > 0.40. The result of the STRING analysis was imported into Cytoscape software (version 3.7.2), and all interaction evidence contributed to nodes was scored with the cutoff as 0.40. Using the cytohubba plugin in Cytoscape, the top 20 nodes were ranked by five algorithms, including MCC, MNC, DMNC, Degree, and the Clustering Coefficient, respectively. The same genes from the five algorithms were tagged as hub genes for subsequent analysis.

### 2.4. Enrichment Analysis

DAVID Bioinformatics Resources 2021 (https://david.ncifcrf.gov/ accessed on 15 September 2023) was used in enrichment analysis including Gene Ontology (GO) annotation analysis and Kyoto Encyclopedia of Genes and Genomes (KEGG) annotation analysis. GO enrichment analysis was employed in three classes of biological processes (BP), molecular functions (MF), and cellular components (CC). In these analyses, GO terms and KEGG pathways were clustered into various groups with different enrichment scores.

Enrichr analysis (https://maayanlab.cloud/Enrichr/ accessed on 15 September 2023) was used to confirm the results of the GO and KEGG from DAVID analysis and identify the gene-related diseases and drugs, using the DisGeNET module and the Proteomics Drug Atlas module, respectively. The results with a *p*-value < 0.05 under the hypergeometric test were considered statistically significant.

### 2.5. Verification of the Hub Genes

The hub genes were verified using two other GSE datasets from the GEO database. The DEGs in GSE6798 (PCOS vs. control) and in GSE76826 (depression vs. control) were separately screened out with the cut-off criteria as *p*-value < 0.05. Then, the overlapped hub genes were identified as the key genes for the comorbidity of PCOS and depression.

### 2.6. Validating Associations of Key Gene Polymorphism and the Comorbidity

In order to validate the key genes found in the above analysis, the data extracted from the UKB database were used. The UKB is a large prospective cohort of over 500,000 participants in the European population and has collected a large amount of phenotypic and genotypic data, including anthropometric information, biochemical and imaging results, and disease diagnosis, as well as lifestyle information. According to the Helsinki Declaration, the UKB obtained written informed consent from all research subjects and ethical approval from the Ethics Committee.

In this study, the women who were with both depression and PCOS from the UKB were selected as the comorbidity case group and healthy women as the control group. The women who had been diagnosed as having depression by doctors or were currently taking antidepressant medication were judged as patients with depression. Similarly, the women being diagnosed as PCOS by doctors or receiving relevant treatment were judged as patients with PCOS.

The inclusion criteria included women: (1) who were aged ≥ 40 years old; (2) who were able to communicate effectively and cooperate during the investigation; and (3) who volunteered to participate in the investigation. The exclusion criteria included women: (1) who were previously diagnosed with neurodegenerative or psychiatric disorders such as anxiety, Parkinson’s disease, and Alzheimer’s disease, and (2) who were unable to complete various examinations and survey questionnaires independently.

Tag SNPs for those key genes were extracted from the European population in the UKB, and the processes included the following: (1) the gene locations were determined through NCBI; (2) info and ped files were generated through the VCF to PED converter in the 1000 Genomes database; (3) tag SNPs were screened using Haploview 4.2 software based on the criteria of Minimum Allele Frequency (MAF) > 0.05 and r^2^ > 0.8; (4) the function of genetic variations for key genes were reviewed from the relevant literature in PubMed.

The Hardy–Weinberg Equilibrium (HWE) test was performed, with *p*-value > 0.05 indicating a genetic balance and good population representativeness. The tolerability (TOL) > 0.1, variance inflation factor (VIF) < 10, and condition index (CI) ≤ 30 indicated no collinearity between independent variables. The relationships of genetic variation between depression and PCOS were analyzed using five genetic models including additive, co-dominant, dominant, recessive, and over-dominant using binary logistic regression analysis, adjusting for covariates such as age, education level, BMI, hypertension, diabetes, and dementia in family history. In addition, the weighted gene score was calculated based on the SNPs in each gene using the weighted gene score method to explore the relationship between the gene score and comorbidity.

### 2.7. Gene-miRNAs Interaction Network Analysis

The key genes were uploaded to the miRWalk database (http://mirwalk.umm.uni-heidelberg.de/ accessed on 20 September 2023) to identify miRNAs that potentially regulate the expression of key genes. The target site of miRNA was in the 3-UTR region. The data of the key gene–miRNA interaction were visualized in Cytoscape. Using the cytohubba plugin to explore the key miRNA, the top 10 nodes were ranked using MCC algorithms.

## 3. Results

### 3.1. Identification of DEGs in PCOS and Depression

**Figure 1:**
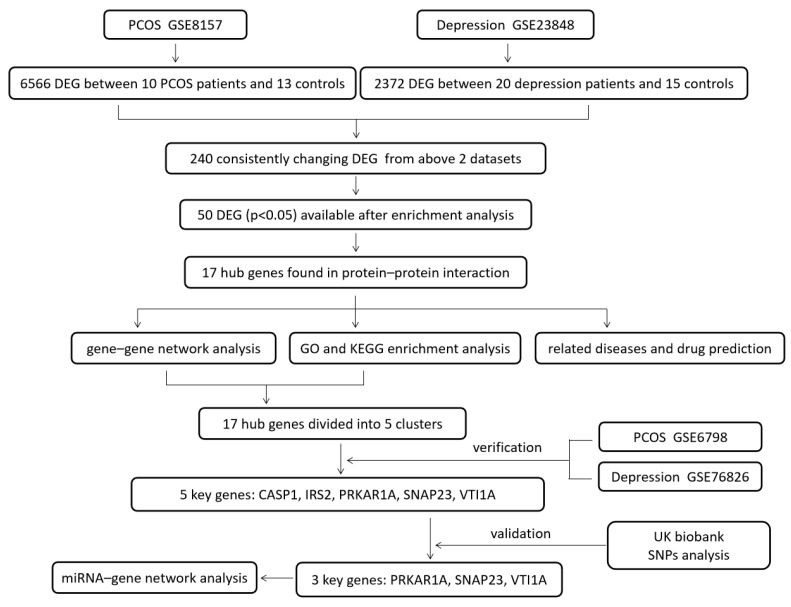
The design of the study.

**Figure 2:**
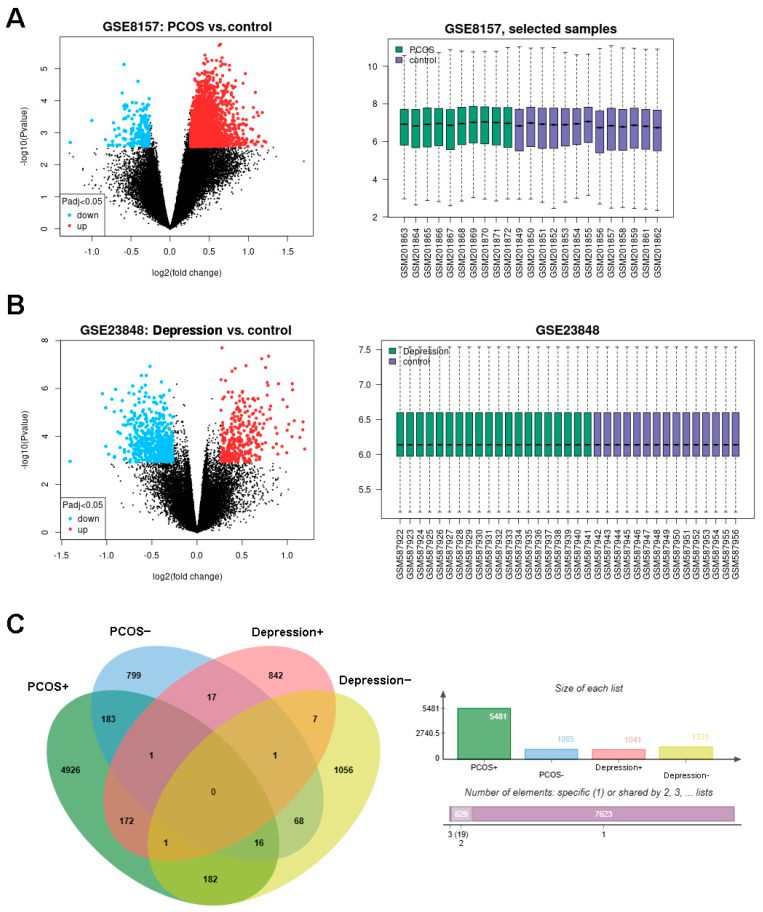
Identification of DEGs in PCOS and depression datasets. (**A**): the DEGs in GSE8157 (PCOS vs. control), (**B**): the DEGs in GSE23848 (depression vs. control), and (**C**): consistently changing DEGs in Venn diagram.

The design of this study is shown in Figure 1. The DEGs of PCOS in the GSE8157 dataset and of depression in the GSE23848 dataset were first screened out, respectively; then, they were used to identify the overlaps, which indicated the potential gene groups associated with the comorbidity of depression and PCOS. A total of 6656 DEGs were found between 10 patients with PCOS and 13 healthy controls, including 5481 up-regulated and 1085 down-regulated DEGs. A list of 2372 DEGs were detected between 20 patients with depression and 15 healthy controls, involving 1041 up-regulated and 1331 down-regulated DEGs. The volcano plots and box line plots of PCOS and depression are separately presented in Figure 2A,B. As shown in the Venn diagram in Figure 2C, 240 overlapped genes were screened out from the two datasets.

### 3.2. PPI Network Construction and Hub Gene Identification

**Figure 3:**
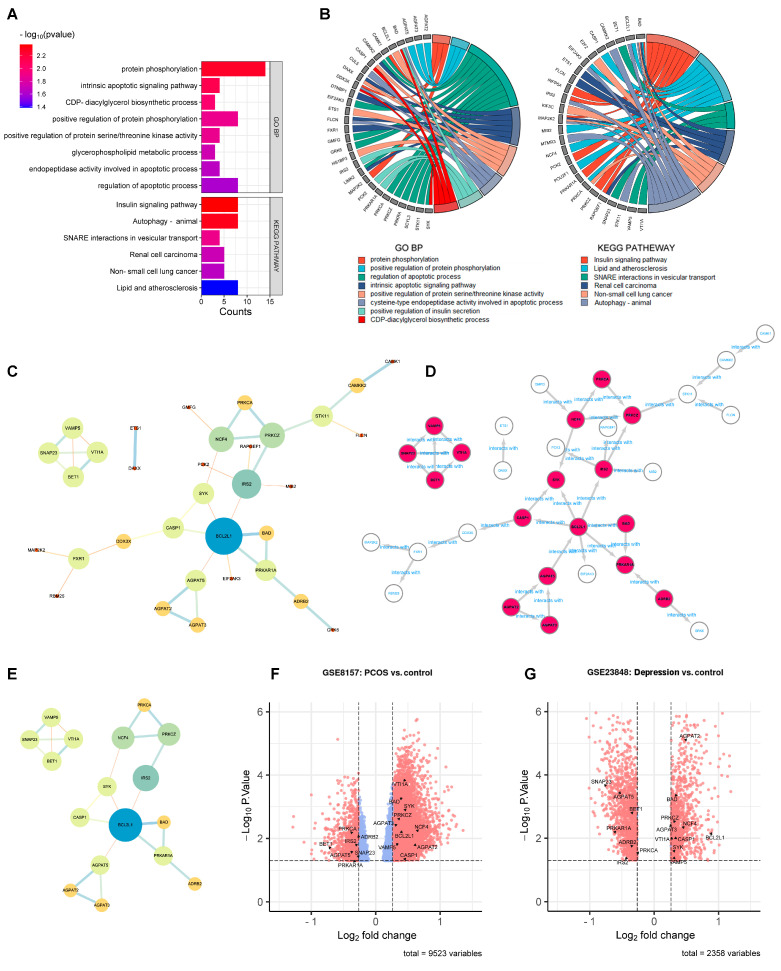
PPI network analysis to identify the hub genes. (**A**): Enrichment analysis conducted before the PPI network analysis. GO terms and KEGG pathways (*p* < 0.05). (**B**): GO terms and KEGG pathways (*p* < 0.05) related genes in enrichment analysis. (**C**): the 50 DEGs found in enrichment analysis. (**D**): the 17 hub genes in PPI network. (**E**): the 17 hub genes. (**F**,**G**): the 17 hub genes marked out in GSE8157 and GES 23848.

Enrichment analysis was conducted before PPI network analysis in order to remove the statistically insignificant GO terms and KEGG pathway-related genes. As shown in Figure 3A,B, the number of DEGs shrunk from 240 to 50 after DAVID analysis. These 50 DEGs were further analyzed by PPI network analysis. As shown in Figure 3C, 33 DEGs with 38 edges remained in the network with all the unconnected nodes removed. The larger size and darker color of the nodes indicated greater values in the network, indicating the more important genes. After calculation using five algorithms, 17 genes with 23 edges were identified as hub genes (Figure 3D,E). The 17 hub genes are marked out in a volcano plot in Figure 3F (GSE8157) and Figure 3G (GES 23848), including 10 up-regulated genes (*AGPAT2*, *AGPAT3*, *BAD*, *BCL2L1*, *CASP1*, *NCF4*, *PRKCZ*, *SYK*, *VAMP5*, and *VTI1A*) and 7 down-regulated genes (*ADRB2*, *AGPAT5*, *BET1*, *IRS2*, *PRKAR1A*, *PRKCA*, and *SNAP23*).

### 3.3. Enrichment Analysis of Hub Genes

**Figure 4:**
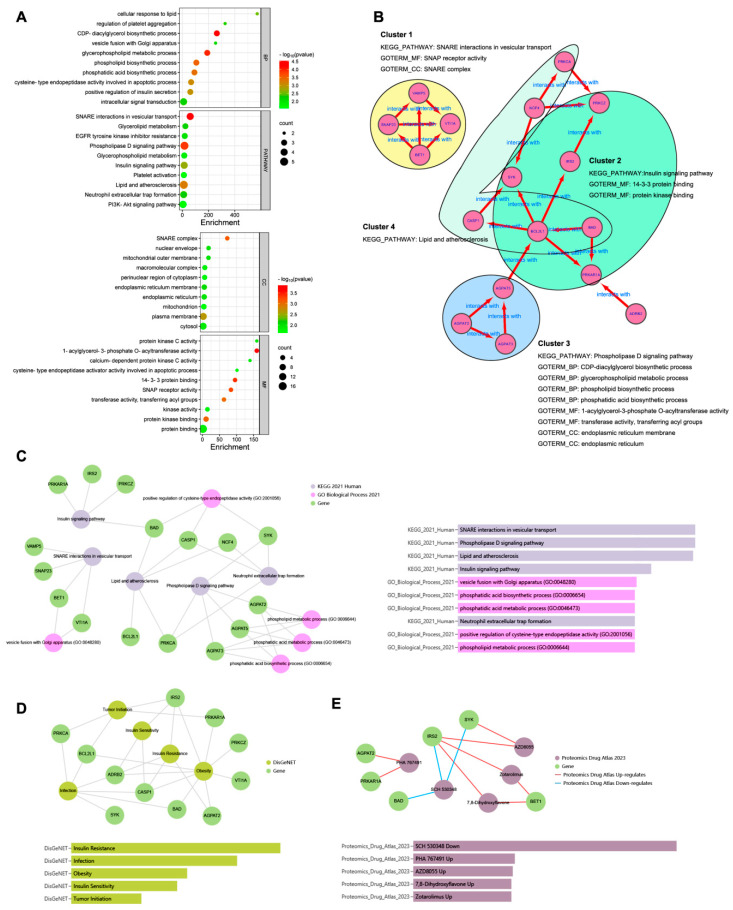
Enrichment analysis of the hub genes. (**A**): the top 10 GO terms classified as BP, CC, and MF and top 10 KEGG pathways in DAVID analysis. (**B**): the 17 hub genes were divided into four clusters. (**C**): the enrichment results of GO BP and KEGG pathway from Enrichr analysis. (**D**): DisGeNET from Enrichr analysis to show the hub gene-related diseases. (**E**): Proteomics Drug Atlas from Enrichr analysis to show the potential drugs.

To explore the biological functions of the 17 hub genes, enrichment analysis was performed. As shown in Figure 4A, the top 10 GO terms classified as BP, CC, and MF and the top 10 KEGG pathways in DAVID analysis were drawn, respectively, in the dot plot. The enrichment results of the GO-BP and KEGG pathway from Enrichr analysis are shown in Figure 4C. The same top significant KEGG pathways, shown in Figure 4A,C, were found to be SNARE interactions in vesicular transport, the phospholipase D signaling pathway, lipid and atherosclerosis, and the insulin signaling pathway. The top GO BP terms shown in Figure 4A,C were not quite the same; however, most belonged to the ancestor tree of the biosynthetic and metabolic processes of glycerophospholipid and phospholipid in QuickGO (www.ebi.ac.uk/QuickGO accessed on 10 September 2023). As Figure 4B shows, the 17 hub genes were divided into four clusters. Each cluster was formed by a group of genes related to one of the KEGG pathways, and their GO-BP, CC, and MF terms combined to indicate that the hub genes exerted their molecular function in specific cellular components in order to achieve the biological processes involving the four KEGG pathways.

In DisGeNET from the Enrichr analysis (Figure 4D), the hub gene-related diseases were shown to be insulin resistance, infection, obesity, insulin sensitivity, and tumor initiation, and the potential drugs from the Proteomics Drug Atlas were listed, such as SCH 530348, PHA 767491, AZD8055, 7,8-dihydroxyflavone, and zotarolimus (Figure 4E).

### 3.4. Verification of the Hub Genes Using Other GSE Datasets

The 17 hub genes screened out were verified using another dataset GSE6798 on PCOS and a dataset GSE76826 on depression. After GEO2R analysis, 10 hub genes were found in GSE6798 and 10 hub genes in GSE76826, respectively. There were five overlapping hub genes in the two datasets including *CASP1*, *IRS2*, *PRKAR1A*, *SNAP23*, and *VTI1A* (Supplementary Table S1), which were identified as the key genes in the comorbidity of PCOS and depression. The five key genes related to three pathways included the SNARE interactions in the vesicular transport pathway (*SNAP23*, *VTI1A*), the insulin signaling pathway (*IRS2*, *PRKAR1A*), and lipid and atherosclerosis (*CASP1*).

### 3.5. Validating the Associations of Key Gene Polymorphism and the Comorbidity

A total of 225 participants (including 45 cases and 180 controls) were included in this study. A list of eight SNPs at *SNAP23*, eight SNPs at *PRKAR1A*, nine SNPs at *VTI1A*, six SNPs at *CASP1*, and eight SNPs at *IRS2* were screened, respectively (listed in Supplementary Table S2). All genetic variation in genotype conformed to the Hardy–Weinberg equilibrium (*p* > 0.05), indicating a good representativeness of the participants. All independent variables conformed to VIF < 10, TOL > 0.1, and CI ≤ 30, showing no multicollinearity existing between the variables.

The results of the key gene polymorphism and comorbidity of PCOS and depression are shown in Table 1. For the *SNAP23* gene, negative associations were found between rs112568544 and the patients with PCOS and depression in the additive genetic model (OR = 0.39, 95% CI: 0.17–0.92), the dominant genetic model (0.34, 0.14–0.83), the co-dominant genetic model (0.32, 0.13–0.80), and the over-dominant genetic model (0.32, 0.13–0.79). A significant relationship was also identified between the weighted gene score of *SNAP23* and the comorbidity (0.61, 0.44–0.83).

For the *PRKAR1A* gene, a negative association was detected between rs11077579 and the patients with PCOS and depression in the co-dominant genetic model (OR = 0.38, 95% CI: 0.14–0.98). Contrary to that, positive associations were discovered between rs4458066 and comorbidity in the additive genetic model (1.78, 1.07–2.97), the recessive genetic model (4.87, 1.39–17.10), and the co-dominant genetic model (5.07, 1.34–19.13). The weighted gene score of *PRKAR1A* was also significantly associated with comorbidity (0.96, 0.93–0.99).

For the *VTI1A* gene, positive associations were shown between rs10885349 and the comorbidity in the co-dominant genetic model (OR = 2.59, 95% CI: 1.06–6.37) and in the over-dominant genetic model (2.59, 1.18–5.67). However, no statistical association was found in the weighted gene scores of *VTI1A* with comorbidity (OR = 1.01, 95% CI: 1.00–1.03).

As for *CASP1* and *IRS2*, no statistically significant association was found between the two gene polymorphisms and comorbidity.

### 3.6. Gene-miRNA Network Construction and Key miRNA Prediction

**Figure 5:**
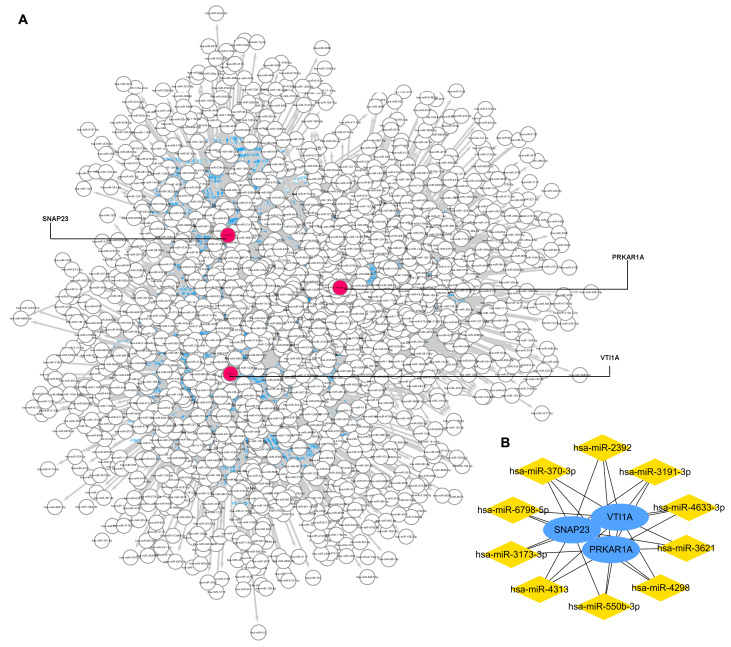
Gene–miRNA interaction network to 3 key genes. (**A**): the gene–miRNA interaction network. (**B**): top 10 miRNAs that regulated the key genes.

The miRWalk database and Cytoscape software were used to identify miRNAs that potentially regulated the three key genes. As Figure 5A shows, the gene–miRNA interaction network was constructed with three key gene nodes and 1483 miRNAs nodes connected to the key genes. The top 10 miRNAs, namely miR-3173-3p, miR-3191-3p, miR-4313, miR-3621, miR-4298, miR-6798-5p, miR-550b-3p, miR-370-3p, miR-2392, and miR-4633-3p, are calculated and visualized in Figure 5B.

## 4. Discussion

Multiple possible associations have been reported from epidemiological and clinical studies between PCOS and depression [4,5,16], indicating the relationship between PCOS and depression to be comorbid. Despite the emerging interest in the last 10 years, the underlying mechanism between this comorbidity remained unclear. In this study aiming to explore the genetic components between PCOS and depression, we found five key genes potentially related to the comorbidity of PCOS and depression through bioinformatic analyses. After validating the associations of key gene polymorphism and the comorbidity, three key genes namely *SNAP23*, *PRKAR1A*, and *VTI1A*, remained significant to the comorbidity.

### 4.1. SNAP23

SNAREs (soluble N-ethylmaleimide-sensitive factor attachment protein receptors), consisting of more than 30 members, is a large family of proteins that plays a major role in intracellular vesicular trafficking through mediating the fusion of donor and acceptor membranes in eukaryotic cells. SNAREs contain one R- and three Q-SNARE (subdivided into Qa, Qb, and Qc) domains. SNAP-25(Synaptosomal-Associated Protein 25) and its paralogs SNAP-23, important subfamily members of SNAREs with the Qb and Qc domains, are specialized for driving regulated exocytosis [17,18,19]. Different from the neuron-specific SNAP-25, SNAP-23 is ubiquitously expressed in the blood and immune system, the musculoskeletal system, most internal organs, secretory and reproductive organs as well as in the brain [17,20]. Matched to its location, SNAP-23 can drive regulated exocytosis of GLUT4 vesicles to the plasma membrane triggered by insulin stimulation in adipocyte and in skeletal muscle [21,22,23], as well as mediate the glutamate release in glia [24,25] and IGF-1 (insulin-like growth factor 1) localization in hippocampal neurons [26,27].

PCOS is a metabolic disorder characterized by profound peripheral insulin resistance, with up to 70% of the patients with PCOS demonstrating insulin resistance [28]. Insulin resistance contributes not only to the metabolic abnormalities in PCOS but also to hyperandrogenemia, one of the diagnostic criteria of PCOS, by stimulating the ovarian androgen production [29,30,31]. There is evidence that insulin stimulates glucose uptake in human skeletal muscle by increasing GLUT4 translocation. In skeletal muscle from insulin resistant individuals, an impaired insulin action on glucose utilization is found to involve the impaired stimulation of GLUT4 translocation, as well as the redistribution of SNAP23 from the plasma membrane to lipid droplets, whether in lean or obese patients [23]. Thus, it is suggested that GLUT4 translocation with SNAP23 redistribution plays a role in insulin resistance in skeletal muscle in patients with PCOS. Not surprisingly, SNAP23 can be screened out as a key gene related to PCOS.

Glutamate is the main excitatory neurotransmitter in the mammalian central nervous system (CNS) and has a major role in the pathophysiology of depression [32,33,34]. It is reported that glutamate release is inhibited through the cleavage of SNAP-23 in satellite glial cells [24]. Glutamate receptor expression is consistent with the highly enriched localization of SNAP-23 on postsynaptic but not presynaptic dendritic spines, which indicates that SNAP-23 can mediate the postsynaptic trafficking of the glutamate receptor [25]. Aside from glutamate, IGF-1 is also widely distributed in the CNS and is known to underlie the pathogenesis of depression [35,36]. SNAP23 is reported to be essential for the exocytosis of plasmalemmal precursor vesicles and the polarized insertion of the IGF-1 receptor [27]. As an important factor both on glutamate release and on IGF-1 localization in CNS, *SNAP23* is evidenced to be closely related to depression. Based on the above, the previous research supports our bioinformatic results that *SNAP23* can be regarded as a key gene in the comorbidity of PCOS and depression.

Furthermore, the results of the SNP genetic analysis indicated that negative associations were found between rs112568544 at *SNAP23* and the patients with PCOS and depression. A significant weighted gene score of *SNAP23* was also presented in this study. All these findings supported *SNAP23* as a key gene for this comorbidity.

### 4.2. VTI1A

VTI (Vacuole Protein Sorting 10-interacting) proteins are a subclass of Qb-SNAREs, including VTI1A and VTI1B, which mediate different steps of endolysosomal trafficking and are necessary for regulated secretion as well [37,38]. VTI1A like SNAP23 is also a component of insulin-sensitive GLUT4-containing vesicles that regulate the GLUT4 trafficking in 3T3-L1 adipocytes [39]. Furthermore, presynaptically but not postsynaptically, the loss of VTI1A impairs spontaneous high-frequency glutamate release in hippocampal neurons, confirming the role of the VTI1A as a key regulator of spontaneous neurotransmission [40]. Thus, for the same reason as *SNAP23*, *VTI1A* is evidenced to be a key gene in the comorbidity of PCOS and depression. *SNAP23* and *VTI1A* are concerned with SNARE interactions in the vesicular transport pathway. Accordingly, SNARE interactions in the vesicular transport pathway that involve *SNAP23* and *VTI1A* is proven to be a vital signaling pathway in the patients with PCOS and depression.

In the genetic analysis, a positive association was found between rs10885349 at *VTI1A* and this comorbidity, which is in line with the above-mentioned results. However, no statistical association was found in the weighted gene scores of *VTI1A*, which implies that *VTI1A* might be a weak key gene for this comorbidity compared to *SNAP23*.

### 4.3. PRKAR1A

PKA (protein kinase A), the main mediator of cAMP signaling pathway, is a second messenger-dependent enzyme essential for various cellular processes such as metabolism, proliferation, differentiation, and apoptosis. PRKAR1A (protein kinase cAMP-dependent type I regulatory subunit α), the main component of type I PKA, regulates the kinase activity in response to cAMP [41]. Deletion of PRKAR1A has been proven to cause embryonic lethality [42]. Snapin, a SNARE-associated protein, is phosphorylated in a cAMP/PKA-dependent manner and interacts with SNAP23 to mediate vesicle fusion and exocytosis ubiquitously in neuronal and non-neuronal cells [43,44]. It is reported that snapin interacts with the exocyst and plays a modulatory role in GLUT4 vesicle trafficking and glucose-stimulated insulin exocytosis [45,46]. In CNS, snapin produces a significant decrease in the uptake activity of the dopamine transporter to mediate dopamine transmission [47]. Various depressive symptoms have been evidenced to be associated with lower dopamine level and the downregulated dopamine transporter in depressed patients [48,49]. Consequently, it is speculated that PRKAR1A mediates PKA-dependent snapin to work together with SNAP23 in the comorbidity of PCOS and depression.

The results of the SNP genetic analysis showed that a negative association in rs11077579, or/and positive associations in rs4458066 at PRKAR1A were related to this comorbidity. A significant weighted gene score of *PRKAR1A* was obtained. These findings indicated that PRKAR1A, as well as *SNAP23*, is a strong key gene for patients with PCOS and depression.

### 4.4. Others

In our study, *IRS2* and *CASP1* were also identified as key genes in the bioinformatic analysis but were not verified successfully by genetic analysis. IRS2 (insulin receptor substrate-2), as well as IRS1, are ubiquitously expressed and are the primary mediators in the insulin-dependent regulation of glucose metabolism in most cells [50]. In response to insulin stimulation, PI3K (phosphotidylinositide-3-kinase) associated with IRS1/IRS2 activates the Akt cascade; then, it subsequently increases the translocation of GLUT4 and SNAP23 mediated fusion [51]. IRS2 might act as an upstream mediator for insulin-dependent GLUT4 translocation and SNARE distribution in muscle and fat cells, and thus connects with PCOS. Different to IRS1, IRS2 plays important roles both in peripheral tissues and in CNS. Accumulating studies have demonstrated that a reduction in intracellular signaling mediated by IGF-1 receptor/IRS2 exerts neuroprotective effects in Alzheimer’s disease [52]. However, little evidence has shown that IRS2 is a cause or an effect of depression until now. Thus, without convincing evidence from the literature and without SNP support, it is still open to discussion as to whether IRS2 can be considered as a key gene for the comorbidity of PCOS and depression.

*CASP1* (caspase-1) plays a fundamental role in innate immunity and in several important inflammatory diseases as the protease activates the pro-inflammatory cytokines proIL-1β and proIL-18 [53]. Pro-inflammatory or inflammatory processes have been strongly implicated in the pathogenesis of both PCOS and depression [54,55]. We tended to speculate that the associations between CASP1, PCOS, and depression were reasonable. However, the result of the SNP genetic analysis did not support CASP1 as a key gene for the comorbidity of PCOS and depression. This might be due to the varieties in the races, the samples of the datasets, or the lack in the literature regarding new SNPs. Further research is needed to explore the relationship between CASP1 and the comorbidity of PCOS and depression.

In the enrichment analysis, many top GO BP terms focused on the biosynthetic and metabolic processes of glycerophospholipid and phospholipid, which related to the phospholipase D signaling pathway. However, we found that the hub genes related to the GO terms and pathways were not verified by other GSE datasets. This might be due to the varieties in the races, samples, or study designs of the different GSE datasets.

In Proteomics Drug Atlas analysis, SCH 530348, PHA 767491, AZD8055, 7,8-dihydroxyflavone, and zotarolimus were listed as potential drugs for the comorbidity. The applications of the potential drugs were checked through the literature but no direct relationships were found between the drugs and the key genes identified in the comorbidity. Further studies are needed to explore this area.

## 5. Conclusions

Despite the emerging interest in the possible causes of PCOS-associated depression in the last 10 years, the underlying mechanisms remained unclear. In this study, for the first time, we used bioinformatic analysis to screen the genetic elements between PCOS and depression.

*SNAP23*, *VTI1A*, and *PRKAR1A* were identified and verified to be key genes related to this comorbidity. We postulated that these three key genes participated directly or indirectly in the imbalanced assembly of the SNARE complex. In CNS, the incorrectly assembled SNAREs mediated the abnormal secretion of neurotransmitters to induce the pathogenesis of depression. Meanwhile in peripheral tissue, the misassembled SNAREs disturbed insulin-sensitive GLUT4 vesicle trafficking and resulted in insulin resistance and metabolic disorders in patients with PCOS. Rs112568544 at *SNAP23*, rs11077579 and rs4458066 at *PRKAR1A*, and rs10885349 at *VTI1A* might be the genetic basis for this comorbidity.

We hope that these findings and their related hypothesis may be helpful to explain the underlying molecular mechanism and provide potential biomarkers for diagnosis and therapy in patients with PCOS suffering from depression. Early intervention may contribute to preventing the pathologies and these key genes may become useful targets to fight the disease.

## 6. Limitations

Three limitations should be noted in our study. First, since few specific biomarkers for PCOS or depression exist in patients, it is difficult and challenging to predict this comorbidity. In the literature analysis, links between the key genes, their targets, and then two diseases are indirect and non-exclusive. Given that the genes in the fields of comorbidity of PCOS and depression are rarely studied, the evidence to support our results is insufficient. Even though we have validated the associations of key genes with this comorbidity using genetic variation data from UKB database, future cohort study is still needed to validate our findings.

Second, in the bioinformatic analysis, we used the GEO2R, STRING, DAVID, and miRWalk tools to screen out the key DEGs, but it was difficult to control the potential confounding factors using these tools. Even though some covariates such as age, education level, BMI, hypertension, diabetes, and dementia in family history were adjusted for genetic models in validating analysis using UKB genetic data, some unknown factors can still affect the associations that we found.

Finally, in our study, the key genes were screened out based on the data from Danish and America population and further validated using data from European population, suggesting that the key genes we identified were more likely to be related to PCOS–depression comorbidity in the Caucasian population. More studies are still needed to verify the roles of the key genes in this comorbidity in other ethnicities.

## Figures and Tables

**Table 1:** SNP genetic analysis to validate the key genes. Rs112568544 at *SNAP23*, rs11077579 and rs4458066 at *PRKAR1A*, and rs10885349 at *VTI1A* were found statistically significant (*p* < 0.05 marked with *) in the comorbidity. The gene score of *SNAP23* and *PRKAR1A* were significant.

Key Gene	SNPs	Model		Disease	No Disease	OR	*p*
SNAP23	rs112568544	additive	CC vs. CA vs. AA	0.3 ± 0.4	0.1 ± 0.3	0.39 (0.17–0.92)	*p* = 0.031 *
		co-dominant	CC	32 (74.4%)	153 (89%)		
			CA	11 (25.6%)	18 (10.5%)	0.32 (0.13–0.80)	*p* = 0.014 *
			AA	0 (0%)	1 (0.6%)	403,884.90 (0.00–Inf)	*p* = 0.988
		dominant	CC	32 (74.4%)	153 (89%)		
			CA + AA	11 (25.6%)	19 (11%)	0.34 (0.14–0.83)	*p* = 0.018 *
		recessive	CC + CA	43 (100%)	171 (99.4%)		
			AA	0 (0%)	1 (0.6%)	507,574.97 (0.00–Inf)	*p* = 0.988
		over-dominant	CC + AA	32 (74.4%)	154 (89.5%)		
			CA	11 (25.6%)	18 (10.5%)	0.32 (0.13–0.79)	*p* = 0.013 *
	Gene_score					0.61 (0.44–0.83)	*p* = 0.002 *
PRKAR1A	rs11077579	additive	CC vs. CT vs. TT	1.1 ± 0.6	1.0 ± 0.8	0.80 (0.50–1.30)	*p* = 0.376
		co-dominant	CC	7 (16.3%)	55 (32%)		
			CT	25 (58.1%)	69 (40.1%)	0.38 (0.14–0.98)	*p* = 0.045 *
			TT	11 (25.6%)	48 (27.9%)	0.58 (0.20–1.70)	*p* = 0.324
		dominant	CC	7 (16.3%)	55 (32%)		
			CT + TT	36 (83.7%)	117 (68%)	0.44 (0.18–1.10)	*p* = 0.078
		recessive	CC + CT	32 (74.4%)	124 (72.1%)		
			TT	11 (25.6%)	48 (27.9%)	1.13 (0.51–2.54)	*p* = 0.759
		over-dominant	CC + TT	18 (41.9%)	103 (59.9%)		
			CT	25 (58.1%)	69 (40.1%)	0.51 (0.25–1.04)	*p* = 0.063
	rs4458066	additive	CC vs. CG vs. GG	0.7 ± 0.6	0.9 ± 0.8	1.78 (1.07–2.97)	*p* = 0.028 *
		co-dominant	CC	17 (39.5%)	57 (32.9%)		
			CG	23 (53.5%)	70 (40.5%)	1.07 (0.50–2.28)	*p* = 0.858
			GG	3 (7%)	46 (26.6%)	5.07 (1.34–19.13)	*p* = 0.017 *
		dominant	CC	17 (39.5%)	57 (32.9%)		
			CG + GG	26 (60.5%)	116 (67.1%)	1.53 (0.74–3.17)	*p* = 0.252
		recessive	CC + CG	40 (93%)	127 (73.4%)		
			GG	3 (7%)	46 (26.6%)	4.87 (1.39–17.10)	*p* = 0.014 *
		over-dominant	CC + GG	20 (46.5%)	103 (59.5%)		
			CG	23 (53.5%)	70 (40.5%)	0.68 (0.34–1.37)	*p* = 0.277
	Gene_score					0.96 (0.93–0.99)	*p* = 0.009 *
VTI1A	rs10885349	additive	AA vs. AC vs. CC	1.0 ± 0.9	1.0 ± 0.7	1.00 (0.63–1.60)	*p* = 0.993
		co-dominant	AA	16 (37.2%)	49 (28.3%)		
			AC	12 (27.9%)	80 (46.2%)	2.59 (1.06–6.37)	*p* = 0.038 *
			CC	15 (34.9%)	44 (25.4%)	1.00 (0.42–2.39)	*p* = 0.994
		dominant	AA	16 (37.2%)	49 (28.3%)		
			AC + CC	27 (62.8%)	124 (71.7%)	1.67 (0.78–3.55)	*p* = 0.186
		recessive	AA + AC	28 (65.1%)	129 (74.6%)		
			CC	15 (34.9%)	44 (25.4%)	0.60 (0.28–1.28)	*p* = 0.189
		over-dominant	AA + CC	31 (72.1%)	93 (53.8%)		
			AC	12 (27.9%)	80 (46.2%)	2.59 (1.18–5.67)	*p* = 0.018 *
	Gene_score					1.01 (1.00–1.03)	*p* = 0.073

## Data Availability

All data generated or analyzed during this study are included in this published article and its supplementary information files.

## Supplementary Materials

The following supporting information can be downloaded at: https://www.mdpi.com/article/10.3390/genes15040494/s1, Table S1: Verify the hub genes with other GSE datasets to identify the key genes. 10 hub genes were found positive in GSE6798 and also 10 hub genes in GSE76826 respectively. The overlapped hub genes in the two datasets were 5 genes including CASP1, IRS2, PRKAR1A, SNAP23, and VTI1A; Table S2: SNPs screened in key genes. 8 SNPs at SNAP23, 8 SNPs at PRKAR1A, 9 SNPs at VTI1A, 6 SNPs at CASP1, and 8 SNPs at IRS2 were screened, respectively.
