# Supplementary material for: Identification of Key Genes and Imbalanced SNAREs Assembly in the Comorbidity of Polycystic Ovary Syndrome and Depression
Source: Genes (Basel). 2024 Apr 15;15(4):494. doi: 10.3390/genes15040494 (PMC11049873; doi:10.3390/genes15040494)
Supplement: Supplementary file 1 [file genes-15-00494-s001.zip › supplymentary table 1.pdf]

Cluster	Hub gene	PCOS GSE6798	Depression GSE76826
Cluster 1	SNAP23	+	+
	BET1		
	VAMP5		+
	VTI1A	+	+
Cluster 2	BAD	+	
	BCL2L1	+	
	IRS2	+	+
	PRKAR1A	+	+
	PRKCZ		+
	SYK		+
Cluster 3	AGPAT2		+
	AGPAT3	+	
	AGPAT5		
Cluster 4	CASP1	+	+
	NCF4		+
	PRKCA	+	
	ADRB2	+	

Supplementary Table S1. Verify the hub genes with other GSE datasets to identify the key genes. 10 hub genes were found positive in GSE6798 and also 10 hub genes in GSE76826 respectively. The overlapped hub genes in the two datasets were 5 genes including *CASP1, IRS2, PRKAR1A, SNAP23*, and *VTI1A*.
