# Supplementary material for: Identification of Key Genes and Imbalanced SNAREs Assembly in the Comorbidity of Polycystic Ovary Syndrome and Depression
Source: Genes (Basel). 2024 Apr 15;15(4):494. doi: 10.3390/genes15040494 (PMC11049873; doi:10.3390/genes15040494)
Supplement: Supplementary file 1 [file genes-15-00494-s001.zip › supplymentary table 2.pdf]

	key Genes				
	SNAP23	*PRKAR1A*	*VTI1A*	*CASP1*	*IRS2*
	rs2595939	rs3744304	rs10885349	rs473549	rs12584130
	rs575723419	rs1112737	rs12780297	rs484626	rs9583424
	rs36090286	rs2909215	rs7099263	rs530537	rs754205
	rs62019288	rs888298	rs11196033	rs1977989	rs754204
SNPs	rs112568544	rs35967565	rs4918773	rs45483194	rs4372569
	rs11630625	rs11077579	rs10787465	rs76705537	rs1967911
	rs33998900	rs4458066	rs6585192		rs4771644
	rs117881887	rs2885731	rs11196120		rs3742210
			rs10787470		

Supplementary Table S2. SNPs screened in key genes. 8 SNPs at *SNAP23*, 8 SNPs at *PRKAR1A*, 9 SNPs at *VTI1A*, 6 SNPs at *CASP1*, and 8 SNPs at *IRS2* were screened, respectively.
